# 

**Published:** 1914-09-19

**Authors:** 


### CONTENTS.

PAGE

**"What can I do to Help my Country?"** ... 663
Defend the Families of Our Sailors and Soldiers.

**Hospital and Institutional News ...** ... ... 665
"The Hospital's" Special Correspondents—The King's Attitude to German Prisoners: A National Contrast—The King's Visit to Netley—An Irish Ambulance Train—The Wages of Panel Practice—Hospital Appeals in Time of War—British Colony's Hospital, Antwerp—Insurance Committees and Soldiers' Dependants—The Besetting Danger of Relief Funds—The First Auxiliary Red Cross Hospital—An Enthusiastic Worker in Cumberland—An Oxford College Closed—Poor-Law Institutions and the War—Infirmary Beds for the Wounded Troops—The "Senior" Red Cross Organisation—Rubber Floors for Hospitals—The Problem of Lighting Emergency Hospitals—Two Practical Lighting Systems—The Medical Treatment of Refugees—The Canadian Military Hospital—Organisation of the Scottish Red Cross—New Address of Uxbridge Cottage Hospital—Women and the Scarcity of Resident Officers—The Patriotism of the Bristol General Hospital—The Week's Drug Market.

**The War and Professional Prospects** ... ... 671
The Outlook for Education and Practice.

**Open-Air Hospitals in War-Time** ... ... 673

By Robert Saundby, M.D., F.R.C.P.

**Reports on Hospitals of the United Kingdom**— 677
By SIR HENRY BURDETT, K.C.B., K.C.V.O.
The Cumberland Infirmary, Carlisle.

[*See over.*]

### CONTENTS—(*continued*).

PAGE

**Soldiers in the Provincial Hospitals** ... ... 675

(*From our Special Correspondents.*)

First Batch Return to Their Depots.

Bristol: Distinguished Visitors — Hartlepools Hospital: From Hospital to Workhouse—Hereford: Terms for Receiving Men—Chatham District: German Sailors—Leicester: Men Return to Depôts—Liverpool: The Base Hospital—Old Perth Infirmary—Sheffield: 3rd Northern General Hospital—Territorials at Tunbridge Wells.

**The After-Histories of Sanatorium Patients...** 679

A Report from Frimley: By a Tuberculosis Officer.

**The National Insurance (Navy and Army) Act, 1914** ... ... ... ... ... ... 680

The Position of Lord Kitchener's Armies.

PAGE

**Round the World** ... ... ... ... ... ... 681

Losses in the Royal Army Medical Corps.
Fellow-Workers and the Roll of Honour.

**The Editor's Letter-Box** ... ... ... ... ... 682

Our Warriors' Dependants and "Relief."
Committees and the Shortage of Resident Medical Officers: Is there Shortage?

**Personalia** ... ... ... ... ... ... 684

**Buildings Contemplated** ... ... ... ... 684

**Institutional Needs** ... ... ... ... ... 684

"Acrosyl."

**The Book Market** ... ... ... ... ... 684

**The Institutional Worker** .. (Suppl.) 1

How to Start a Hospital Laundry.
Ladies' Linen League in Time of War.
War Matters.
Questions for August and September.

